# Small secreted proteins enable biofilm development in the cyanobacterium *Synechococcus elongatus*

**DOI:** 10.1038/srep32209

**Published:** 2016-08-25

**Authors:** Rami Parnasa, Elad Nagar, Eleonora Sendersky, Ziv Reich, Ryan Simkovsky, Susan Golden, Rakefet Schwarz

**Affiliations:** 1 The Mina and Everard Goodman Faculty of Life Sciences, Bar-Ilan University, Ramat-Gan, 5290002, Israel; 2 Department of Biological Chemistry, Weizmann Institute of Science, Rehovot, 7610001, Israel; 3 Division of Biological Sciences, University of California, San Diego, La Jolla, CA 92093, USA

## Abstract

Small proteins characterized by a double-glycine (GG) secretion motif, typical of secreted bacterial antibiotics, are encoded by the genomes of diverse cyanobacteria, but their functions have not been investigated to date. Using a biofilm-forming mutant of *Synechococcus elongatus* PCC 7942 and a mutational approach, we demonstrate the involvement of four small secreted proteins and their GG-secretion motifs in biofilm development. These proteins are denoted EbfG1-4 (enable biofilm formation with a GG-motif). Furthermore, the conserved cysteine of the peptidase domain of the Synpcc7942_1133 gene product (dubbed PteB for peptidase transporter essential for biofilm) is crucial for biofilm development and is required for efficient secretion of the GG-motif containing proteins. Transcriptional profiling of *ebfG*1-4 indicated elevated transcript levels in the biofilm-forming mutant compared to wild type (WT). However, these transcripts decreased, acutely but transiently, when the mutant was cultured in extracellular fluids from a WT culture, and biofilm formation was inhibited. We propose that WT cells secrete inhibitor(s) that suppress transcription of *ebfG*1-4, whereas secretion of the inhibitor(s) is impaired in the biofilm-forming mutant, leading to synthesis and secretion of EbfG1-4 and supporting the formation of biofilms.

Cyanobacteria in nature often reside in biofilms, bacterial assemblages encased in a self-produced extracellular matrix^1^,^2^. Such microbial assemblages can lead to economic loss, for example due to material decay or blockage of flow through membranes in desalination plants^3^,^4^. However, phototrophic biofilms may be beneficial in other processes, for example when employed in wastewater purification systems and bioremediation processes^5^,^6^,^7^,^8^. Additionally, biofilm-based biofuel production systems that are efficient to harvest have been shown to generate high product yields with minimal water and nutrient inputs^9^,^10^. Furthermore, since grazing by small protistan predators imposes a major difficulty in growing cyanobacteria in open ponds, the biofilm, which serves as a physical barrier against these predators, has the potential for crop protection^11^,^12^,^13^.

In comparison with vast knowledge on the molecular mechanisms that underlie biofilm development in heterotrophic bacteria (see reviews^14^,^15^,^16^,^17^,^18^,^19^,^20^,^21^,^22^,^23^,^24^,^25^ and references therein), relatively little is known about these processes in cyanobacteria. Recent studies, however, have provided new insight into cyanobacterial aggregation processes and biofilm development^26^,^27^,^28^,^29^,^30^. For example, inactivation of *Synechocystis* PCC 6803 homologs of ATP binding cassette (ABC) transporters causes flocculation of the cultures and adherence to glass culture tubes^30^. An exoprotein of *Anabaena* sp. PCC 7120 is required for filament adhesion and aggregation^31^. Involvement of the signaling molecule cyclic dimeric GMP (c-di-GMP) in biofilm formation has been shown for *Synechocystis*^26^ and *Thermosynechococcus elongatus*^32^. In the latter case, blue light, acting through the cyanobacteriochrome SesA, elevates c-di-GMP and triggers a sessile phenotype at 31 °C, a relatively low temperature for *T. elongatus*^32^. SesA, together with two additional cyanobacteriochromes, provides a color-sensitive system for c-di-GMP-dependent cell aggregation^27^. Constitutive expression of the c-di-GMP producing enzyme, diguanylate cylase, in *Synechocystis* results in biofilm formation, whereas expression of phosphodiesterase to reduce c-di-GMP causes enhanced buoyancy^26^.

We recently demonstrated that the constitutive planktonic growth of the cyanobacterium *Synechococcus elongatus* PCC 7942 under standard laboratory conditions is due to a self-suppression mechanism that relies on secreted inhibitor(s)^33^. Here, we identify genes that enable biofilm development and provide evidence for the extracellular presence of four gene products denoted EbfG1-4 (enable biofilm formation possessing a double-glycine motif). Using a mutational approach, we demonstrate the requirement of the peptidase domain of the Synpcc7942_1133 product for efficient secretion of EbfG1-4. These data, together with transcriptional analysis of *ebfG1*-*4*, indicate that mediation of biofilm development in *S. elongatus* relies on extracellular inhibitors that repress expression of extracellular components required for biofilm formation.

## Results

### Small proteins characterized by a double glycine motif enable biofilm development

**Figure 1:**
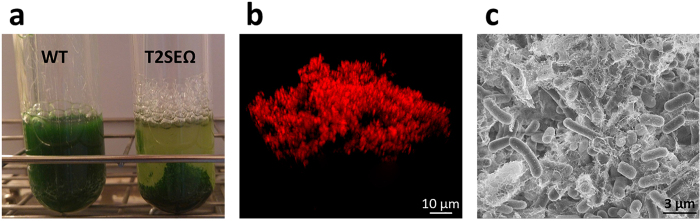
The T2SE-mutant (T2SEΩ) forms biofilms. (**a**) Cultures of WT and T2SEΩ (7 days old). (**b,c**) Biofilms of T2SEΩ imaged by fluorescence microscopy and cryo-SEM, respectively.

Previously, we demonstrated that inactivation of genes encoding homologs of type II protein secretion systems or the type IV pilus assembly apparatus (components of these complexes share a high degree of similarity^34^,^35^,^36^,^37^) impairs a biofilm inhibitory mechanism of *S. elongatus* and enables biofilm development^33^. Inactivation of synpcc7942_2071, which encodes a homolog of subunit E of type II secretion systems (T2SE), results in a mutant (T2SEΩ) that adheres to the growth tube in contrast to the planktonic phenotype of the WT strain (Fig. 1a). Analyses by fluorescence microscopy (Fig. 1b) and cryo-scanning electron microscopy (cryo-SEM, Fig. 1c) substantiate biofilm development by T2SEΩ (see Schatz *et al*.^33^ for additional cryo-SEM as well as environmental-SEM analyses).

**Figure 2:**
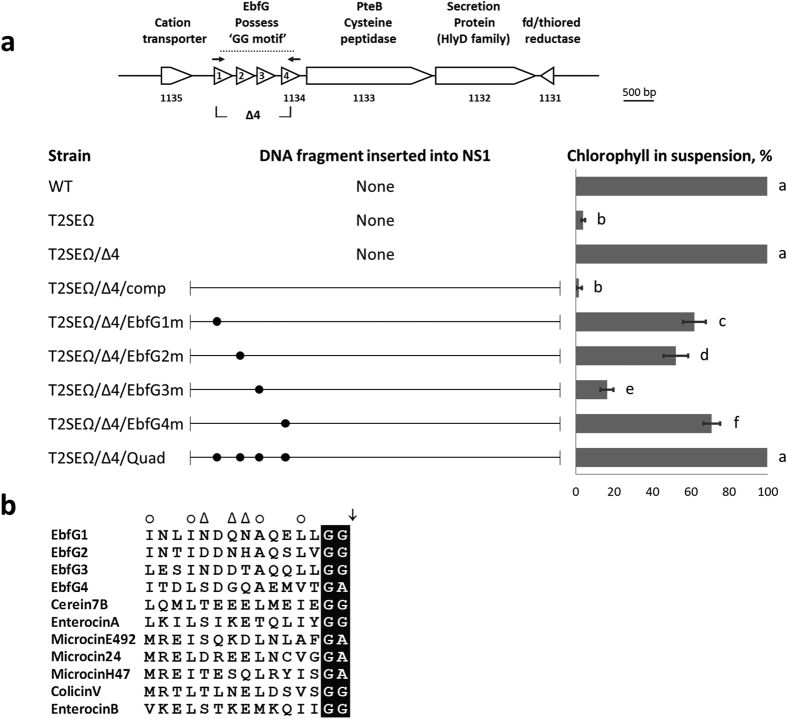
Small proteins characterized by double glycine motifs are required for biofilm development. (**a**) Genomic region of *ebfG*1-4, genes whose products are characterized by a double-glycine secretion motif. Deletion of these four genes is indicated by ∆4. Arrows indicate PCR primers used for detection of the four-gene transcript (see Fig. 3b). Bar graph presents percentage of total chlorophyll in the suspended cells (average of three independent biological repeats ± standard deviation). Strains analyzed include: wild type (WT), the biofilm-forming mutant T2SEΩ, and deletion of *ebfG*1-4 in T2SEΩ (T2SEΩ/∆4). /comp designates addition into neutral site (NS) 1 of the indicated DNA fragment, covering *ebfG*1 through Synpcc7942_1131, for complementation experiments (see T2SEΩ/∆4/comp). Similar fragments into which mutations changing the conserved GG-secretion motif to double alanine were introduced into the T2SEΩ/∆4 strain (for example, T2SEΩ/∆4/EbfG1m). Each motif conversion is indicated by a dot positioned at the cognate ORF in the genomic map. The ‘Quad’ fragment contains motif conversions in all four proteins. For statistical analysis we employed analysis of variance (ANOVA) and post analysis multiple comparison contrasts. In the bar graph, different letters assign statistical significance (see Table S1 for p-values). (**b**) Sequence of the double-glycine motif of EbfG1-4 and selected secreted peptides. Cerein7B of *Bacillus cereus*, EnterocinA and EnterocinB of *Enterococcus faceum*, MicrocinE492 of *Klebsiella pneumonia*, and Microcin24, MicrocinH47, and ColicinV of *Escherichia coli*. Black shading indicates the double-glycine or glycine-alanine present just prior to the peptide cleavage site (arrow). Positions typically occupied by hydrophobic or hydrophilic amino acids are indicated by a circle or a triangle, respectively.

We previously demonstrated that the gene Synpcc7942_1134 is required for biofilm formation by *S. elongatus*^33^. The product of this gene exhibits an N-terminal secretion motif (denoted GG-motif) typical of bacterial antibiotics, *e.g.* microcins. Such proteins undergo maturation during secretion by cleavage of the secretion motif after a conserved glycine-glycine (or glycine-alanine, as in the case of Synpcc7942_1134) motif 38,39. Wang *et al*.^40^ identified three previously unannotated open reading frames located immediately upstream of Synpcc7942_1134, the putative products of which are also characterized by a GG-motif^40^ (also see Fig. 2a,b). These gene products are referred to here as EbfG1-4. The putative precursors encoded by these genes are approximately 9–10 kDa, with 6–7 kDa remaining after their maturation by removal of the secretion motif^40^. Proteins possessing GG-motifs are prevalent in cyanobacteria^40^; however, mature EbfG1-4 of *S. elongatus* do not show homology to proteins from other cyanobacteria or exhibit domains of known function.

To test whether the GG-motifs of EbfG1-4 are required for biofilm development we combined mutations in these loci with mutation of Synpcc7942_2071 (T2SEΩ strain). The T2SEΩ mutation alone results in a culture in which about 5% of the total chlorophyll is present in suspended cells (Fig. 2a), while the remaining chlorophyll resides in biofilm-forming cells attached to the bottom and sides of the culture vessel. Deletion of *ebfG*1-4 in the context of the T2SEΩ mutation completely abolished biofilm formation, with 100% of the chlorophyll present in suspended cells, similar to the WT (Fig. 2a, T2SEΩ/Δ4). Recombination of a DNA fragment bearing the genes *ebfG*1-4 into the genome at neutral site 1 (NS1) complements the phenotype of T2SEΩ/Δ4, restoring biofilm formation to the level observed in T2SEΩ (Fig. 2a, T2SEΩ/Δ4/comp).

To examine whether the GG-motifs in EbfG1-4 are required for biofilm formation, we altered the *trans-*complementing DNA fragment to separately modify each GG-motif to AA. For example, introduction of DNA encoding a modified EbfG1 GG-motif (with EbfG2-4 sequences unchanged) only partially restored biofilm development to the completely planktonic T2SEΩ/Δ4 strain (Fig. 2a, T2SEΩ/Δ4/EbfG1m). On average, 60% of the total chlorophyll was found in suspended cells, significantly more than the 2–5% found inT2SEΩ or T2SEΩ/∆4/comp (see Table S1 for p-values). Similarly, modification of the GG-motif of EbfG2 or EbfG4 to AA (Fig. 2a, T2SEΩ/Δ4/EbfG2m and T2SEΩ/Δ4/EbfG4m, respectively) in the complementing DNA fragment partially restored biofilm development to the T2SEΩ/Δ4 strain (50–70% chlorophyll in suspended cells). In contrast, mutation of the GG-motif in EbfG3 only slightly interfered with biofilm development; in this case just 15% of the chlorophyll was in the planktonic cells (Fig. 2a, compare T2SEΩ/Δ4/EbfG3m and T2SEΩ/∆4/comp). When the GG-motifs in all four proteins were mutated simultaneously, biofilm development was completely abolished (Fig. 2a, T2SEΩ/Δ4/Quad). In summary, these data support the suggestion that each of the GG-motifs contributes to biofilm formation. Furthermore, because the combination of the four mutations completely abrogated biofilm development, whereas each individual mutation only partially interfered with biofilm formation, we suggest functional redundancy between EbfG1-4.

### Transcripts of ebfG1-4 are highly abundant in T2SEΩ

**Figure 3:**
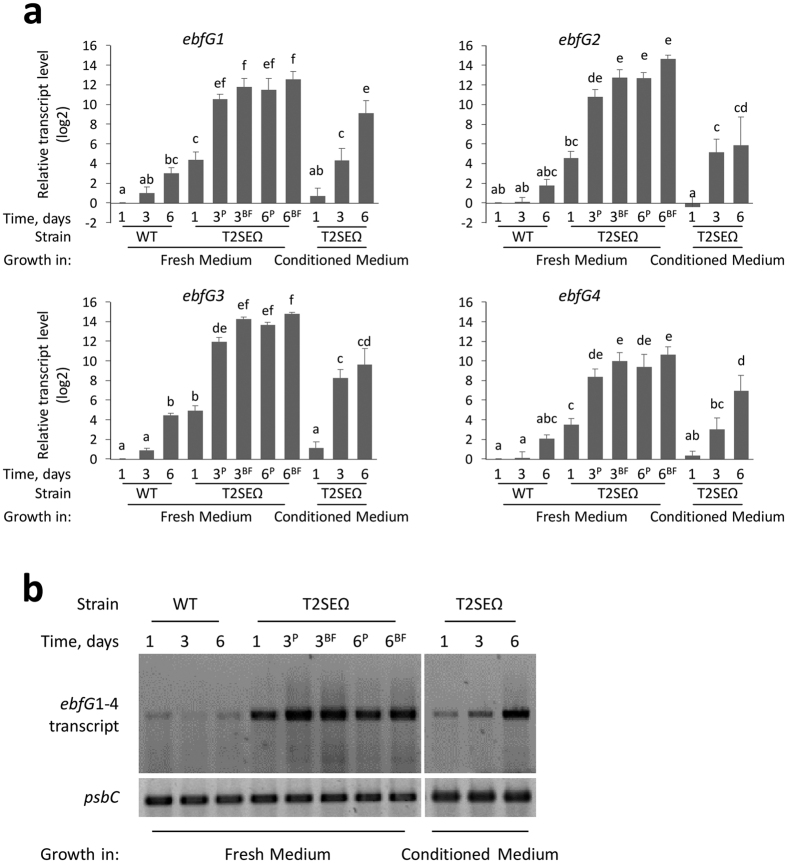
Transcript level of *ebfG1-4* is elevated in T2SEΩ compared to WT, and is reduced under conditioned medium from a WT culture. (**a**) RT-qPCR analyses of *ebfG*1-4 in cells grown for 1, 3 and 6 days. Cultures were initiated in fresh medium or conditioned medium from a WT culture. When biofilms were present (T2SEΩ grown in fresh medium for 3 and 6 days), the transcript level is shown for planktonic cells (3^P^, 6^P^) and cells in the biofilm (3^BF^, 6^BF^). Transcript levels of *psbC*, encoding CP43, a chlorophyll binding protein of photosystem II, served to normalize total RNA levels. Transcript abundance is indicated as fold change relative to WT on the first day of growth in fresh medium. Data are presented on a log2 scale. Bars indicate average of three independent biological repeats ± standard deviation). For statistical analysis we employed three factors ANOVA and post analysis multiple comparison contrasts. In each bar graph, different letters assign statistical significance (see Table S2 for p-values). Similar trends in transcript level were previously reported for *ebfG4* using semiquantitative RT-qPCR^33^. (**b**) Gel image of PCR products representing a four-gene polycistronic transcript of *ebfG1-4*. Primers used for the PCR are indicated in Fig. 2a and Table S4.

Differences in transcript levels from the *ebfG*1-4 genes between the WT and T2SEΩ were followed using RT-qPCR. Initially, T2SEΩ grows planktonically; however, biofilms start forming, usually on the third day of growth. Transcript levels of *ebfG*1-4 in T2SEΩ 1 day after inoculation into fresh medium were 14–37 fold higher compared to WT (Fig. 3a, day 1 Fresh Medium). Once biofilms formed, the sessile as well as the planktonic cells of the mutant were characterized by significantly higher levels of these transcripts compared to 1 day old mutant cultures (Fig. 3a, T2SEΩ Fresh Medium). The fact that transcripts of *ebfG*1-4 are similarly elevated in planktonic and biofilm forming cells of T2SEΩ at 3 and 6 days (Fig. 3a, 3^P^, 3^BF^, 6^P^, 6^BF^) suggests that high transcript levels of these genes are not by themselves sufficient to drive biofilm development.

Extracellular fluids from a WT culture (hereafter, conditioned medium) inhibit biofilm development of T2SEΩ^33^. Inoculation of the T2SEΩ mutant into conditioned medium significantly reduced *ebfG*1-4 transcript levels during the first day of growth, as compared to T2SEΩ at the same growth stage in fresh medium. Further growth of T2SEΩ in conditioned medium resulted in increased transcript levels of *ebfG*1-4 compared to day 1, but did not reach the levels detected in biofilm forming cells of this mutant (Fig. 3a). In two cases, *ebfG1* and *ebfG4*, the transcript levels after 6 days were not significantly different between planktonic T2SEΩ cells grown in fresh medium and those grown in conditioned medium, indicating that conditioned medium results in expression levels of these genes consistent with planktonic cells but not with biofilm forming cells.

The similar trends of transcriptional changes of *ebfG*1-4 suggested that these genes are co-regulated. Using the primers indicated in Fig. 2a for RT-PCR, we detected a polycistronic transcript containing *ebfG*1-4 (Fig. 3b). Likely due to low expression in WT, this transcript was not previously identified in RNA-seq of *S. elongatus* by Vijayan *et al*.^41^. The mechanism underlying regulation of these genes is yet unknown.

### A cysteine peptidase is required for efficient secretion of EbfG1-4

**Figure 4:**
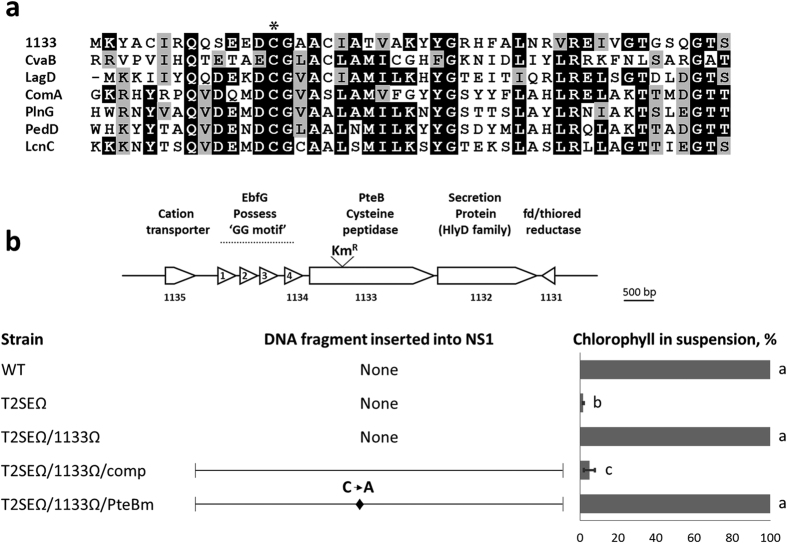
The conserved cysteine in the peptidase domain of PteB is essential for biofilm development. (**a**) Amino acid alignment of the N-terminal domain of C39 peptidases and the product of Synpcc7942_1133 from *S. elongatus*. CvaB of *Escherichia coli*, LagD and LcnC of *Lactococcus lactis*, ComA of *Streptococcus pneumonia*, PlnG of *Lactobacillus plantarum* and PedD of *Pediococcus acidilactici*. Identity or similarity between at least 50% of the aligned sequences is indicated by black or grey shading, respectively. The asterisk denotes the conserved cysteine residue typically involved in processing of a double-glycine containing substrate. (**b**) A kanamycin resistance cassette (Km^R^) was inserted into Synpcc7942_1133 and combined with inactivation of *t2sE* (T2SEΩ/1133Ω). /comp designates addition into neutral site (NS) 1of the indicated DNA fragment, covering *ebfG*1 through Synpcc7942_1131, for complementation experiments (see T2SEΩ/1133Ω/comp). A similar fragment into which a mutation changing the conserved cysteine to alanine (indicated by a diamond) was introduced into the T2SEΩ/1133Ω strain (T2SEΩ/1133Ω/PteBm). Bar graphs indicate percentage of chlorophyll in suspended cells (average of three independent biological repeats ± standard deviation). For statistical analysis we employed ANOVA and post analysis multiple comparison contrasts. In the bar graph, different letters assign statistical significance (see Table S3 for p-values).

Using insertional inactivation, we previously demonstrated that the gene Synpcc7942_1133 is essential for biofilm development^33^. The protein encoded by this gene is characterized by a peptidase domain of the ‘C39 family’ (Fig. 4a), named after the conserved cysteine residue essential for transport and maturation of precursor substrates that possess a GG-motif^42^,^43^. Akin to the analysis performed with the GG-motifs, a mutational complementation approach was employed to examine the requirement for a functional peptidase domain to enable biofilm development. The double mutant T2SEΩ/1133Ω exhibits a planktonic phenotype^33^ (Fig. 4b). Biofilm development was restored when a DNA fragment bearing Synpcc7942_1133 was combined with the T2SEΩ/1133Ω double mutation (Fig. 4b, T2SEΩ/1133Ω/comp). Replacement of the conserved cysteine residue with alanine in this *trans*-complementing DNA fragment, however, completely abolished biofilm formation (Fig. 4b, T2SEΩ/1133Ω/PteBm), in agreement with the hypothesis that the protein encoded by Synpcc7942_1133 (denoted PteB for peptidase transporter essential for biofilm) is involved in the secretion of EbfG1-4.

**Figure 5:**
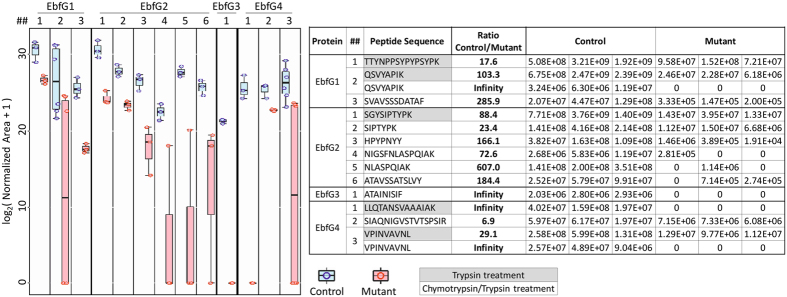
The conserved cysteine in the peptidase domain of PteB is required for efficient secretion of EbfG1-4, small proteins exhibiting a double glycine motif. Mass spectrometry analyses of conditioned media from 2 d old cultures of the control strain (T2SEΩ/1133Ω/comp) and the mutant, in which the conserved cysteine in the peptidase domain was mutated to alanine (T2SEΩ/1133Ω/PteBm). Samples were digested prior to analyses using trypsin alone or in combination with chymotrypsin. Table provides normalized areas of detected peptides for three independent biological replicates. Normalized area is proportional to the peptide level. Zero values indicate the inability to detect a peptide. Graph on the left presents the log_2_(t + 1) transformation of the data for each peptide (indicated by protein and number) as box plots, with the box representing the second and third quartiles, the horizontal line indicating the median, and the whisker bars representing the maximum and minimum values. Each individual data point is plotted as a circle (Control = blue, Mutant = red). Note that on the box plot of the mutant, the bottom dot is a zero value, which actually represents multiple observation as is indicated in the table. The differences between the control and the mutant strain for peptide EbfG1#2 and EbfG4#3 are significant at a p-value of 0.039 and for all other peptides at a p-value of 0.05, as calculated using a one-tailed Mann-Whitney test for two independent samples.

To test whether PteB is required for secretion of the small GG-motif proteins of *S. elongatus*, we analyzed the extracellular fluids of the strains T2SEΩ/1133Ω/comp and T2SEΩ/1133Ω/PteBm for the presence of these secreted proteins. The control strain T2SEΩ/1133Ω/comp forms biofilms similarly to T2SEΩ and likely has high transcript levels for *ebfG*1-4. Comparative analyses by mass spectrometry indicated significantly higher levels of peptides derived from EbfG1-4 in the conditioned media of the biofilm forming strain T2SEΩ/1133Ω/comp as compared to that of the planktonic strain T2SEΩ/1133Ω/PteBm, which possesses the cysteine to alanine mutation (Fig. 5). The presence of some of the peptides in extracellular fluids from T2SEΩ/1133Ω/PteBm cultures may be due to cell lysis. It is also possible that the cysteine to alanine mutation does not completely abrogate PteB function and some secretion and maturation does occur in the mutant. This potential activity, however, is insufficient to support biofilm development because the T2SEΩ/1133Ω/PteBm grows planktonically (Fig. 4b).

Of note, the peptides TTYNPPSYPYPSYPK (EbfG1), SGYSIPTYPK (EbfG2) and LLQTANSVAAAIAK (EbfG4) were detected by mass spectrometry in conditioned media. Unlike all other peptides detected in these analyses, which represent cleavage by the trypsin or chymotrypsin peptidases used for sample digestion prior to analysis, these three peptides likely represent cleavage of the secretion motif, because in the putative precursor protein they are preceded by GG (EbfG1 and EbfG2) or GA in the case of EbfG4 (see Fig. S1) and not by an amino acid typically preceding trypsin or chymotrypsin cleavage sites. These data provide support for maturation and secretion of these small proteins by removal of the secretion motif. In summary, the mutational approach and the mass spectrometry analyses support the suggestion that efficient secretion of EbfG1-4 is required for biofilm development, and that the peptidase domain of PteB is involved in the secretion process.

## Discussion

The data suggest that *S. elongatus* employs a GG-secretion motif, typically associated with microcins, and a transport system involving a cysteine peptidase for secretion and maturation of the small proteins encoded by *ebfG*1-4. Wang *et al*. identified similar gene clusters throughout the vast diversity of sequenced cyanobacterial genomes and suggested that these gene products represent a potential source for natural products^40^. To our knowledge, the function of these small cyanobacterial proteins has not been experimentally examined prior to this work. This study, which implicates the small proteins of *S. elongatus* in biofilm development, assigns a novel function to proteins possessing a microcin-like secretion motif.

Cyanobacterial proteins with a microcin-like secretion motif may represent a large variety of functions. In this vein, it should be noted that HetC of *Anabaena* sp. PCC 7120, which is required for normal differentiation of heterocysts, exhibits a domain organization similar to the cysteine peptidase PteB. It was suggested that HetC may be involved in processing of PatS, the small protein that is required for normal heterocyst pattern formation^44^, although PatS is not characterized by a GG-secretion motif.

**Figure 6:**
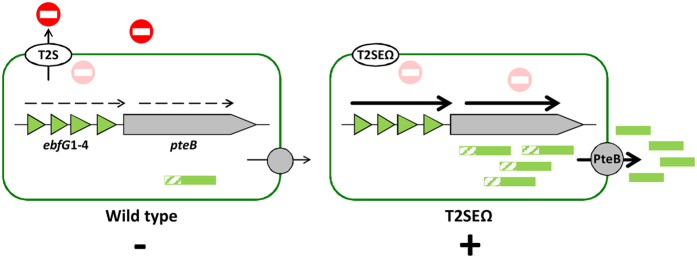
Transcription of *ebfG*1-4 and secretion of the small proteins encoded by these genes is governed by extracellular biofilm inhibitor(s). Using a T2S-like system, WT cells secrete an inhibitor (red) which suppresses transcription of the genes *ebfG*1-4 (this study) and *pteB*^33^. The biofilm-forming mutant, T2SEΩ, is most likely impaired in secretion of the inhibitory factor, and therefore expresses ‘biofilm-genes’ at a higher level. Consequently, the small proteins that possess GG-secretion motif are produced at increased levels compared to the WT and are secreted and processed by a transport system in which the cysteine peptidase, PteB takes part. Dashed and thick arrows represent low and high transcript levels, respectively. The hatched regions of the secreted proteins represent the GG-motif. Biofilm development or its absence is indicated by plus (+) or minus (−) symbols, respectively. The T2S system may not be directly involved in secretion of the inhibitor.

Our experiments revealed that transcript abundance of *ebfG*1–4, which are cotranscribed, is highly elevated in the biofilm forming strain T2SEΩ as compared to WT (Fig. 3). Inoculation of the T2SEΩ mutant in conditioned medium from a WT culture prevents biofilm formation^33^ and substantially reduces the transcript levels of *ebfG*1-4 at the initial stage of growth (Fig. 3 day 1 and day 3). These data, together with the results supporting secretion of EbfG1-4 and involvement of PteB in secretion, allow us to refine our previous model for biofilm development by *S. elongatus* (Fig. 6). Using a T2S-like system, the WT deposits a biofilm inhibitor to the extracellular milieu and consequently represses transcription of *ebfG*1-4 (Fig. 3) and *pteB* (see data for Synpcc7942_1133^33^). Suppression of the expression of these genes, even transiently, was associated with biofilm inhibition. In T2SEΩ, secretion of the biofilm inhibitor is impaired, allowing high levels of transcription of *ebfG*1-4 (Fig. 3) and *pteB*^33^ and the small proteins encoded by *ebfG*1-4 are synthesized at high levels. Adequate secretion and maturation of EbfG1-4 by a transport system in which the cysteine peptidase (PteB) takes part, is required for biofilm formation.

## Methods

### Strains, culture conditions, biofilm quantification, and microscopy

*Synechococcus elongatus* PCC 7942 and all derived strains were grown essentially as described previously^33^. Specifically, 25 ml cultures were grown in round-bottom Pyrex tubes (20 cm in length, 3 cm in diameter). Cotton-plugged Pasteur pipette (23 cm long) inserted through a sponge-plug served for bubbling of 3% CO_2_ in air into the cultures. The tip of the Pasteur pipette was placed 1–2 cm above the bottom of the tube. Prior to bubbling into the culture, the gas was humidified by passing through a bottle with double distilled water and filtered using 0.22 μm filter. BG11 medium^45^ served for culturing; however, to reproducibly observe biofilm formation, it was important to add the ferric ammonium citrate and citric acid components (final concentrations 0.0226 and 0.0312 mM, respectively) from freshly made stocks. Autoclaved BG11 was used within 4 days to inoculate cultures. Cultures were grown at 30 °C and under incandescent light (20–30 μmol photons m^−2^ sec^−1^).

For assessment of biofilm formation, cells were cultured under continuous bubbling. Experiments were initiated by diluting cultures at the exponential phase of growth to an optical density at 750 nm of 0.5. Biofilm development under this setting typically initiated following 2–3 days of growth and quantification was performed after 7 days to allow assessment of fully developed biofilms (longer growth time did not increase biofilm development). Percentage of chlorophyll in suspended cells served to quantity biofilm formation as follows. The suspended fraction was sampled for chlorophyll determination by extraction in 80% acetone (final concentration). In cases were the suspended fraction appeared especially dense (planktonic strains or poor biofilm formers), a 0.2 mL sample was used for extraction. When robust biofilms were formed, 15 mL of planktonic cells were removed and concentrated 3 to 6-fold by centrifugation (5000 g, 10 min) prior the chlorophyll extraction in 80% acetone. Determination of chlorophyll in the biofilm was performed following removal of the planktonic cells using a pipette and addition of 80% acetone to the sessile cells. Extraction was carried out over-night in the refrigerator. Dilution in 80% acetone was performed to reach the linear range of the calibration curve and chlorophyll was quantified based on absorbance at 663 nm. For statistical analysis of comparisons of chlorophyll in suspension we employed analysis of variance (ANOVA) and post analysis multiple comparison contrasts (see statistical grouping in Figs 2 and 4 and Tables S1 and S3 for p-values).

For collection of conditioned medium, cultures were centrifuged (5000 g, 10 min) at room temperature, and the supernatant was removed and passed through a 0.22 μm filter. Supplementation with nutrients by addition of medium stock solutions as in the preparation of fresh growth medium was performed when the conditioned medium served for cyanobacterial growth.

For microscopic analysis of biofilms, microscope slides were inserted into growth tubes containing T2SEΩ cultures (for 6 days) so that biofilms formed on the slides. A Leica TCS SPE DM2500 was employed for imaging by fluorescence microscopy using objective HCX APO l 40x/0.80 (excitation 532 nm, emission 620–650 nm). 3D image reconstruction was performed using Imaris. Analysis by SEM was described previously^33^.

### Genetic manipulations of cyanobacterial cells

Insertional inactivation of genes Synpcc7942_2071 (*t2sE*), Synpcc7942_1134 (*ebfG4*), and Synpcc7942_1133 was described previously^33^. In the case of deletion of *ebfG1* through *ebfG4* (∆4), disruption was obtained by deletion of the fragment between the *NheI* and *ClaI* sites. Resulting constructs were introduced into *S. elongatus* using standard transformation methods that take advantage of its natural competence^46^. Mutant cyanobacterial clones resistant to the appropriate antibiotic were confirmed for double homologous recombination (allele replacement) and complete chromosomal segregation using PCR on genomic DNA. Primers and additional cloning information are provided in Table S4. Inactivation of *t2SE* impaired the natural DNA competence of *S. elongatus*; thus, to obtain T2SEΩ strains with additional genetic changes, we initially introduced the other genetic modifications and subsequently inactivated *t2sE*.

Vectors used to introduce the WT and mutated complementation DNA fragments into NS1 were generated using the GeneArt^®^ Seamless Cloning and Assembly Kit (Life Technologies) on PCR-generated cloning fragments and standardized devices (a chloramphenicol resistance device and a NS1 device with tetracycline resistance) derived via ZraI or *Eco*RV-HF (New England Biolabs) restriction digestion of CYANO-VECTOR donor plasmids as described by Taton *et al*.^47^. To improve the efficiency of cloning, the 6-kb WT DNA fragment was constructed from two 3-kb fragments amplified with the Q5^®^ High-Fidelity DNA polymerase (New England Biolabs). To introduce glycine- or cysteine to alanine mutations, the appropriate cloning fragment was assembled from subfragments; these segments were generated using seamless assembly cloning primers designed to introduce the appropriate base mutations at the center of the overlap between subfragments. Combining multiple such subfragments or generating subfragments from previously mutated vectors allowed the generation of the quadruple GG-mutation vector. The sequence of each vector was confirmed. These vectors were used to transform *S. elongatus* strains as described above. For additional information see Table S4 and Fig. S2.

### Mass spectrometry analysis

Conditioned medium was harvested as described above. Mass spectrometry was performed by the de Botton Institute for Protein Profiling at The Nancy and Stephen Grand Israel National Center for Personalized Medicine (Weizmann Institute of Science).

#### Sample preparation

Culture supernatants were concentrated ~20 fold on 3 kDa molecular weight cutoff-filters. Proteins were reduced by incubation with dithiothreitol (5 mM; Sigma) for 45 min at 60 °C, and alkylated with 10 mM iodoacetamide (Sigma) in the dark for 45 min at 21 °C. Proteins were then subjected to digestion with trypsin (Promega; Madison, WI, USA, ratio of 50:1 protein amount:enzyme amount) for 4 h at 37 °C followed by digestion with chymotrypsin (Sigma, ratio of 50:1 protein amount:enzyme amount) for 16 h at 37 °C. The digestions were stopped by trifluroacetic acid (1%). Following digestion, peptides were desalted using solid-phase extraction columns (Oasis HLB, Waters, Milford, MA, USA). The samples were stored in −80 °C until further analysis.

#### Liquid chromatography

ULC/MS grade solvents were used for all chromatographic steps. Each sample was loaded using split-less nano-Ultra Performance Liquid Chromatography (10 kpsi nanoAcquity; Waters, Milford, MA, USA). The mobile phase was: A) H_2_O + 0.1% formic acid and B) acetonitrile +0.1% formic acid. Desalting of the samples was performed online using a reversed-phase C18 trapping column (180 μm internal diameter, 20 mm length, 5 μm particle size; Waters). The peptides were then separated using a T3 HSS nano-column (75 μm internal diameter, 250 mm length, 1.8 μm particle size; Waters) at 0.35 μL/min. Peptides were eluted from the column into the mass spectrometer using the following gradient: 4% to 30% B in A (vol/vol) in 105 min, 35% to 90% B in A in 5 min, maintained at 95% for 5 min and then back to initial conditions.

#### Mass Spectrometry

The nano Ultra Performance Liquid Chromatography was coupled online through a nanoESI emitter (10 μm tip; New Objective; Woburn, MA, USA) to a quadrupole orbitrap mass spectrometer (Q Exactive Plus, Thermo Scientific) using a FlexIon nanospray apparatus (Proxeon). For initial identification, data were acquired in Data-Dependent Acquisition (DDA) mode, using a Top20 method. MS1 resolution was set to 70,000 (at 400 m/z) and maximum injection time was set to 20 msec. MS2 resolution was set to 17,500 and maximum injection time of 60 msec. For targeted analysis, data was acquired in Parallel Reaction Monitoring (PRM) mode, monitoring previously identified peptides from EbfG1-4. MS2 resolution was set to 35,000 (at 400 m/z) and maximum injection time of 100 msec.

#### Data processing and analysis

For identification purposes, raw data was first processed using Proteome Discoverer v1.41. MS/MS spectra were searched using Mascot v2.4 (Matrix Sciences) and Sequest HT. Data were searched against the *S. elongatus* protein database as downloaded from UniprotKB (http://www.uniprot.org/), appended with EbfG1-4, along with 125 common laboratory contaminant proteins. Fixed modification was set to carbamidomethylation of cysteines and variable modification was set to oxidation of methionines. Search results were then imported back to Expressions to annotate identified peaks. Proteins were then grouped based on shared peptides and Identifications were filtered such that the global false discovery rate was maximum of 1%. For PRM analysis, raw data and DDA search results were imported into the Skyline software (https://skyline.gs.washington.edu/). The software was used for retention time alignment, peak detection of peptide fragments and their quantification.

Statistical analysis used the Mann-Whitney U test, a non-parametric test, because the majority of the data (or any viable transformation of it) are not normally distributed.

### RNA preparation and RT-PCR

RNA was prepared as previously described^48^ and treated with DNase (TURBO DNase, Ambion). Random hexamers (Promega) were used to prime cDNA using 1.5 μg RNA and reverse transcriptase (RevertAid, Fermentas). PCR amplification was performed using Fast SYBR Green Master Mix (Thermo Fisher Scientific) with addition of betaine pH 9.0 (final concentration of 0.525M) and a CFX96 Touch Real-Time PCR Detection System (Bio-Rad). Melting curve analysis was performed (65 to 95 °C, 0.5 °C/5s) to confirm amplification of a single cDNA sequence for each gene. Primer dimers or unexpected amplicons were not observed. No signals were detected in the negative controls (samples in which reverse transcriptase was not added). Specific primers for RT-qPCR and RT-PCR of the *ebfG*1-4 transcript are indicated in Table S4. Transcript levels of *psbC*, encoding CP43, a chlorophyll binding protein of photosystem II, served to normalize total RNA levels.

For statistical analysis we employed three factor ANOVA to calculate the covariance (normality and equality of variances were tested using Kruskal-Wallis & Levene’s tests respectively, both found to be >0.05). Next, we used multiple comparison contrasts to evaluate the differences of each pair of comparison. In each bar graph in Fig. 3a, different letters assign statistical significance (see Table S2 for p-values).

## Additional Information

**How to cite this article**: Parnasa, R. *et al*. Small secreted proteins enable biofilm development in the cyanobacterium *Synechococcus elongatus. Sci. Rep.* **6**, 32209; doi: 10.1038/srep32209 (2016).

## Supplementary Material

Supplementary Information
