# Supplementary material for: Small secreted proteins enable biofilm development in the cyanobacterium Synechococcus elongatus
Source: Sci Rep. 2016 Aug 25;6:32209. doi: 10.1038/srep32209 (PMC4997328; doi:10.1038/srep32209)
Supplement: Supplementary Information [file srep32209-s1.pdf]

Small secreted proteins enable biofilm development in the cyanobacterium *Synechococcus elongatus*

Rami Parnasa[a], Elad Nagar[a,] , Eleonora Sendersky[a], Ziv Reich[b], Ryan Simkovsky[c], Susan Golden[c] and Rakefet Schwarz[a*]

[a]The Mina and Everard Goodman Faculty of Life Sciences, Bar-Ilan University, Ramat-Gan, 5290002, Israel.

[b]Department of Biological Chemistry, Weizmann Institute of Science, Rehovot, 7610001, Israel.

[c]Division of Biological Sciences, University of California, San Diego, La Jolla, CA 92093, USA.

[*]Corresponding author.

Running title: Small secreted proteins enable biofilms

Supplementary information

p-value grouping	variants	WT	T2SEΩ	T2SEΩ/Δ4	T2SEΩ/Δ4/ comp	T2SEΩ/Δ4/ EbfG1m	T2SEΩ/Δ4/ EbfG2m	T2SEΩ/Δ4/ EbfG3m	T2SEΩ/Δ4/ EbfG4m	T2SEΩ/Δ4/ Quad
a	WT	1.000	2.50E-11	1.000	2.50E-11	2.50E-11	2.50E-11	2.50E-11	2.50E-11	1.000
b	T2SEΩ	2.50E-11	1.000	2.50E-11	1.000	2.50E-11	2.50E-11	7.80E-08	2.50E-11	2.50E-11
a	T2SEΩ/Δ4	1.000	2.50E-11	1.000	2.50E-11	2.50E-11	2.50E-11	2.50E-11	2.50E-11	1.000
b	T2SEΩ/Δ4/comp	2.50E-11	1.000	2.50E-11	1.000	2.50E-11	2.50E-11	1.32E-07	2.50E-11	2.50E-11
c	T2SEΩ/Δ4/EbfG1m	2.50E-11	2.50E-11	2.50E-11	2.50E-11	1.000	6.30E-04	2.50E-11	1.81E-03	2.50E-11
d	T2SEΩ/Δ4/EbfG2m	2.50E-11	2.50E-11	2.50E-11	2.50E-11	6.30E-04	1.000	2.50E-11	3.26E-11	2.50E-11
e	T2SEΩ/Δ4/EbfG3m	2.50E-11	7.80E-08	2.50E-11	1.32E-07	2.50E-11	2.50E-11	1.000	2.50E-11	2.50E-11
f	T2SEΩ/Δ4/EbfG4m	2.50E-11	2.50E-11	2.50E-11	2.50E-11	1.81E-03	3.26E-11	2.50E-11	1.000	2.50E-11
a	T2SEΩ/Δ4/Quad	1.000	2.50E-11	1.000	2.50E-11	2.50E-11	2.50E-11	2.50E-11	2.50E-11	1.000

Table S1: p-values for GG-mutations.

ebfG1

			Fresh Medium								Conditioned Medium		
p-value groupin		variants	WT 1d	WT 3d	WT 6d	T2SEΩ 1d	T2SEΩ 3d^{P}	T2SEΩ 3d^{BF}	T2SEΩ 6d^{P}	T2SEΩ 6d^{BF}	T2SEΩ 1d	T2SEΩ 3d	T2SEΩ 6d
a	Fresh Medium	WT 1d	1.000	0.520	2.21E-03	7.96E-04	1.00E-10	7.32E-13	2.43E-12	2.64E-13	0.396	4.54E-07	1.87E-08
ab		WT 3d	0.520	1.000	0.012	4.84E-03	7.17E-10	4.56E-12	1.57E-11	1.60E-12	0.837	3.62E-06	1.47E-07
bc		WT 6d	2.21E-03	0.012	1.000	0.720	2.87E-06	1.25E-08	4.86E-08	3.91E-09	0.020	8.65E-03	5.45E-04
c		T2SEΩ 1d	7.96E-04	4.84E-03	0.720	1.000	9.05E-06	3.92E-08	1.54E-07	1.21E-08	0.008	0.021	1.54E-03
ef		T2SEΩ 3d^{P}	1.00E-10	7.17E-10	2.87E-06	9.05E-06	1.000	0.096	0.209	0.044	1.36E-09	0.010	0.098
f		T2SEΩ 3d^{BF}	7.32E-13	4.56E-12	1.25E-08	3.92E-08	0.096	1.000	0.670	0.713	8.28E-12	8.00E-05	1.55E-03
ef		T2SEΩ 6d^{P}	2.43E-12	1.57E-11	4.86E-08	1.54E-07	0.209	0.670	1.000	0.429	2.89E-11	2.93E-04	5.02E-03
f		T2SEΩ 6d^{BF}	2.64E-13	1.60E-12	3.91E-09	1.21E-08	0.044	0.713	0.429	1.000	2.87E-12	2.53E-05	5.35E-04
ab	Conditioned Medium	T2SEΩ 1d	0.396	0.837	0.020	8.33E-03	1.36E-09	8.28E-12	2.89E-11	2.87E-12	1.000	7.01E-06	2.86E-07
c		T2SEΩ 3d	4.54E-07	3.62E-06	8.65E-03	0.021	1.02E-02	8.00E-05	2.93E-04	2.53E-05	7.01E-06	1.000	0.322
e		T2SEΩ 6d	1.87E-08	1.47E-07	5.45E-04	1.54E-03	0.098	1.55E-03	5.02E-03	5.35E-04	2.86E-07	0.322	1.000

ebfG2

			Fresh Medium								Conditioned Medium		
p-value groupin		variants	WT 1d	WT 3d	WT 6d	T2SEΩ 1d	T2SEΩ 3d^{P}	T2SEΩ 3d^{BF}	T2SEΩ 6d^{P}	T2SEΩ 6d^{BF}	T2SEΩ 1d	T2SEΩ 3d	T2SEΩ 6d
ab	Fresh Medium	WT 1d	1.000	0.948	0.471	0.069	7.59E-05	5.80E-06	6.61E-06	4.94E-07	0.868	0.040	0.020
ab		WT 3d	0.948	1.000	0.511	0.079	9.29E-05	7.14E-06	8.14E-06	6.09E-07	0.818	0.046	0.024
abc		WT 6d	0.471	0.511	1.000	0.261	6.76E-04	5.71E-05	6.49E-05	5.06E-06	0.376	0.170	0.098
bc		T2SEΩ 1d	0.069	0.079	0.261	1.000	0.015	1.68E-03	1.89E-03	1.76E-04	0.049	0.797	0.583
de		T2SEΩ 3d^{P}	7.59E-05	9.29E-05	6.76E-04	0.015	1.000	0.417	0.440	0.119	4.54E-05	0.027	0.052
e		T2SEΩ 3d^{BF}	5.80E-06	7.14E-06	5.71E-05	1.68E-03	0.417	1.000	0.967	0.445	3.41E-06	3.43E-03	7.48E-03
e		T2SEΩ 6d^{P}	6.61E-06	8.14E-06	6.49E-05	1.89E-03	0.440	0.967	1.000	0.421	3.89E-06	3.84E-03	8.32E-03
e		T2SEΩ 6d^{BF}	4.94E-07	6.09E-07	5.06E-06	1.76E-04	0.119	0.445	0.421	1.000	2.90E-07	3.82E-04	9.04E-04
a	Conditioned Medium	T2SEΩ 1d	0.868	0.818	0.376	0.049	4.54E-05	3.41E-06	3.89E-06	2.90E-07	1.000	0.027	0.013
c		T2SEΩ 3d	0.040	0.046	0.170	0.797	0.027	3.43E-03	3.84E-03	3.82E-04	0.027	1.000	0.770
cd		T2SEΩ 6d	0.020	0.024	0.098	0.583	0.052	7.48E-03	8.32E-03	9.04E-04	0.013	0.770	1.000

ebfG3			Fresh Medium								Conditioned Medium		
p-value groupin		variants	WT 1d	WT 3d	WT 6d	T2SEΩ 1d	T2SEΩ 3d^{P}	T2SEΩ 3d^{BF}	T2SEΩ 6d^{P}	T2SEΩ 6d^{BF}	T2SEΩ 1d	T2SEΩ 3d	T2SEΩ 6d
a	Fresh Medium	WT 1d	1.000	0.520	2.21E-03	7.96E-04	1.00E-10	7.32E-13	2.43E-12	2.64E-13	0.396	4.54E-07	1.87E-08
a		WT 3d	0.520	1.000	0.012	4.84E-03	7.17E-10	4.56E-12	1.57E-11	1.60E-12	0.837	3.62E-06	1.47E-07
b		WT 6d	2.21E-03	0.012	1.000	0.720	2.87E-06	1.25E-08	4.86E-08	3.91E-09	0.020	8.65E-03	5.45E-04
b		T2SEΩ 1d	7.96E-04	4.84E-03	0.720	1.000	9.05E-06	3.92E-08	1.54E-07	1.21E-08	8.33E-03	0.021	1.54E-03
de		T2SEΩ 3d^{P}	1.00E-10	7.17E-10	2.87E-06	9.05E-06	1.000	0.096	0.209	0.044	1.36E-09	0.010	0.098
ef		T2SEΩ 3d^{BF}	7.32E-13	4.56E-12	1.25E-08	3.92E-08	0.096	1.000	0.670	0.713	8.28E-12	8.00E-05	1.55E-03
ef		T2SEΩ 6d^{P}	2.43E-12	1.57E-11	4.86E-08	1.54E-07	0.209	0.670	1.000	0.429	2.89E-11	2.93E-04	5.02E-03
f		T2SEΩ 6d^{BF}	2.64E-13	1.60E-12	3.91E-09	1.21E-08	0.044	0.713	0.429	1.000	2.87E-12	2.53E-05	5.35E-04
a	Conditioned Medium	T2SEΩ 1d	0.396	0.837	0.020	8.33E-03	1.36E-09	8.28E-12	2.89E-11	2.87E-12	1.000	7.01E-06	2.86E-07
c		T2SEΩ 3d	4.54E-07	3.62E-06	8.65E-03	0.021	0.010	8.00E-05	2.93E-04	2.53E-05	7.01E-06	1.000	0.322
cd		T2SEΩ 6d	1.87E-08	1.47E-07	5.45E-04	1.54E-03	0.098	1.55E-03	5.02E-03	5.35E-04	2.86E-07	0.322	1.000

ebfG4			Fresh Medium								Conditioned Medium		
p-value groupin		variants	WT 1d	WT 3d	WT 6d	T2SEΩ 1d	T2SEΩ 3d^{P}	T2SEΩ 3d^{BF}	T2SEΩ 6d^{P}	T2SEΩ 6d^{BF}	T2SEΩ 1d	T2SEΩ 3d	T2SEΩ 6d
a	Fresh Medium	WT 1d	1.000	0.904	0.135	0.014	2.88E-07	7.31E-09	2.61E-08	1.67E-09	0.789	0.032	8.78E-06
a		WT 3d	0.904	1.000	0.168	0.018	4.25E-07	1.07E-08	3.84E-08	2.43E-09	0.884	0.041	1.29E-05
abc		WT 6d	0.135	0.168	1.000	0.298	3.69E-05	9.40E-07	3.42E-06	2.06E-07	0.215	0.486	9.28E-04
c		T2SEΩ 1d	0.014	0.018	0.298	1.000	8.85E-04	2.67E-05	9.35E-05	5.97E-06	0.026	0.727	0.016
de		T2SEΩ 3d^{P}	2.88E-07	4.25E-07	3.69E-05	8.85E-04	1.000	0.254	0.455	0.110	6.81E-07	3.15E-04	0.291
e		T2SEΩ 3d^{BF}	7.31E-09	1.07E-08	9.40E-07	2.67E-05	0.254	1.000	0.688	0.637	1.70E-08	8.82E-06	0.032
de		T2SEΩ 6d^{P}	2.61E-08	3.84E-08	3.42E-06	9.35E-05	0.455	0.688	1.000	0.385	6.13E-08	3.15E-05	0.076
e		T2SEΩ 6d^{BF}	1.67E-09	2.43E-09	2.06E-07	5.97E-06	0.110	0.637	0.385	1.000	3.84E-09	1.95E-06	0.010
ab	Conditioned Medium	T2SEΩ 1d	0.789	0.884	0.215	0.026	6.81E-07	1.70E-08	6.13E-08	3.84E-09	1.000	0.057	2.05E-05
bc		T2SEΩ 3d	0.032	0.041	0.486	0.727	3.15E-04	8.82E-06	3.15E-05	1.95E-06	0.057	1.000	6.40E-03
d		T2SEΩ 6d	8.78E-06	1.29E-05	9.28E-04	0.016	0.291	0.032	0.076	0.010	2.05E-05	6.40E-03	1.000

Table S2: P-values of comparisons of transcript level of *ebfG*1-4 in wild type (WT) and T2SEΩ. Green shading indicates p-values smaller than 0.05.

p-value grouping	variants	WT	T2SEΩ	T2SEΩ/ 1133Ω	T2SEΩ/ 1133Ω/ comp	T2SEΩ/ 1133Ω/ PteBm
a	WT	1.000	4.64E-13	1.000	4.64E-13	1.000
b	T2SEΩ	4.60E-13	1.000	4.64E-13	6.92E-06	4.64E-13
a	T2SEΩ/1133Ω	1.000	4.64E-13	1.000	4.64E-13	1.000
c	T2SEΩ/1133Ω/comp	4.60E-13	6.90E-06	4.60E-13	1.000	4.60E-13
a	T2SEΩ/1133Ω/PteBm	1.000	4.64E-13	1.000	4.64E-13	1.000

Table S3: p-values for C39A

**Gene disruption**	**Primer sequence**	**Insertion of spc cassette**	
Deletion of *ebfG*1-4 (Δ4)	ATGAGTTCCAACGAAGCCCT<br>ATCAAGTGCGACGCAGCTAT	*Cla*I *Nhe*I	
**RT-qPCR**	**Primer sequence**	**PCR efficiency**	**Tm(˚C)**
ebfG1	CTACCTACAATCCACCGAGC<br>AGCGTCAGAACTACTAACTGC	96%	74.79 ± 0.35
ebfG2	ATCGAGCGTTCAGCAGTCG<br>CATAGACAAGTGAAGTGGC	97%	76.94 ± 0.17
ebfG3	ACTGCTCAACAGCTCTTGG<br>GAGATGTCACACTCTGACC	92%	73.40 ± 0.21
ebfG4	GAGGGCATCGATATGCAAGT<br>TGCTGCTGCTACTGAATTGG	101%	74.17 ± 0.30
psbC	GCACCTTCTTCAGCCAAGAC<br>ATGGCCTTAAAGACCAGCAAG	95%	74.81 ± 0.30

**Primers used to detect the *ebfG*1-4 transcript**	**Primer sequence**	
ebfG1 forward	ATGCTCAGGAACTTCTAGGC	
*ebfG*4 reverse	AAGATTGACAGCTACGTT	
**Complementation Vectors**	**Primer sequence**	**Purpose**
RP10-C2G-F	atcccgccgccgccgccggatTGTACTGC GACTCGACCAAG	Fragment generation
RP10-R3	CTAGCTTGAGTGCTCCCCAAG	Fragment generation
RP10seq5	CTTGGGGAGCACTCAAGC	Fragment generation and sequencing
RP10-G5C5-R	atccccccgggggcccccgatGTTATGAA CGTTGGCGATCG	Fragment generation
ORF3GGmutAAF<br>(used to generatre EbfG1m)	CAGGAACTTCTAGcCGcAACTA CCTACAATC	Fragment generation
ORF3GGmutAAR<br>(used to generatre EbfG1m)	GATTGTAGGTAGTTgCGgCTAG AAGTTCCTG	Fragment generation

Complementation Vectors	Primer sequence	Purpose
ORF2GGmutAAF<br>(used to generatre EbfG2m)	CAATCCCTTGTGGcAGCgAGTGGTTATTC	Fragment generation
ORF2GGmutAAR<br>(used to generatre EbfG2m)	GAATAACCACTCgCTgCCACAAGGGATTG	Fragment generation
ORF1GGmutAAF<br>(used to generatre EbfG3m)	CTCAACAGCTCTTGGcCGcaAAATTCTG	Fragment generation
ORF1GGmutAAR<br>(used to generatre EbfG3m)	CAGAATTTtgCGgCCAAGAGCTGTTGAG	Fragment generation
1134GAmutAA-F<br>(used to generatre EbfG4m)	CTGAGATGGTGACAGcAGCATTATTGCAAAC	Fragment generation
1134GAmutAA-R<br>(used to generatre EbfG4m)	GTTTGCAATAATGCTgCTGTCACCATCTCAG	Fragment generation
1133CmutA-F<br>(used to generate PteBm)	CAAAGTGAAGAGGACgcTGGAGCGGCCTGCATC	Fragment generation
1133CmutA-R<br>(used to generate PteBm)	GATGCAGGCCGCTCCAgcGTCCTCTTCACTTTG	Fragment generation
RP10seq1	GTATCAACAGGGACACCAGG	Sequencing
RP10seq2	GCAATTTCTGGAGCTACATCG	Sequencing
RP10seq3	GCAGTTACAGCCTAACCAAAAAC	Sequencing
RP10seq4	CATTGCAGACCCAGGGC	Sequencing
RP10seq6	CCGTCATGGAAAGTCAGATCC	Sequencing
RP10seq7	AGTTCTGGGATTCATTGGGAC	Sequencing
RP10seq8	TCCAAACCCGTCGAATAATGC	Sequencing
NS1-F (see reference 49)	CGTCGAAGATGGAAAAGCTC	Sequencing
pRP10<br>(used to generate comp and T2SEΩ/1133Ω/comp)	TGTACTGCGACTCGACCAAG<br>GTTATGAACGTTGGCGATCG	Fragment generation

Table S4: Summary of molecular manipulations. The primers used to remove *ebfG*1-4 served for PCR and cloning of a DNA fragment in which the fragment between the *Cla*I and *Nhe*I sites was deleted and replaced with a spectinomycin cassette (spc). The same primers served to confirm complete segregation. For primers used to generate complementation vectors, sequences that match the vector backbone or generate a mutation

in the genomic DNA-derived fragment are in lowercase. For RT-qPCR analyses, average (± standard deviation) of melting temperature (Tm) is indicated.

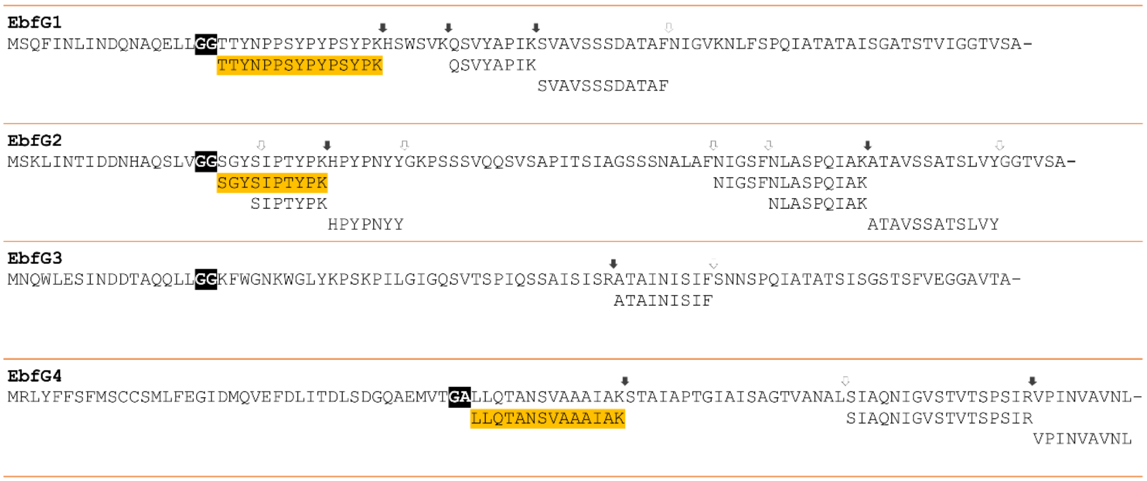

Figure S1: Amino acid sequences of EbfG1-4. Peptides detected by mass spectrometry are shown below each cognate protein. Black and white arrows point at trypsin and chymotrypsin cleavage sites, respectively. Black shading indicates the two glycine residues (glycine and alanine in case of EbfG4; also see Fig. 1b) of the GG-motif. Highlighted in orange are peptides that likely represent maturation by cleavage after the GG-motif.

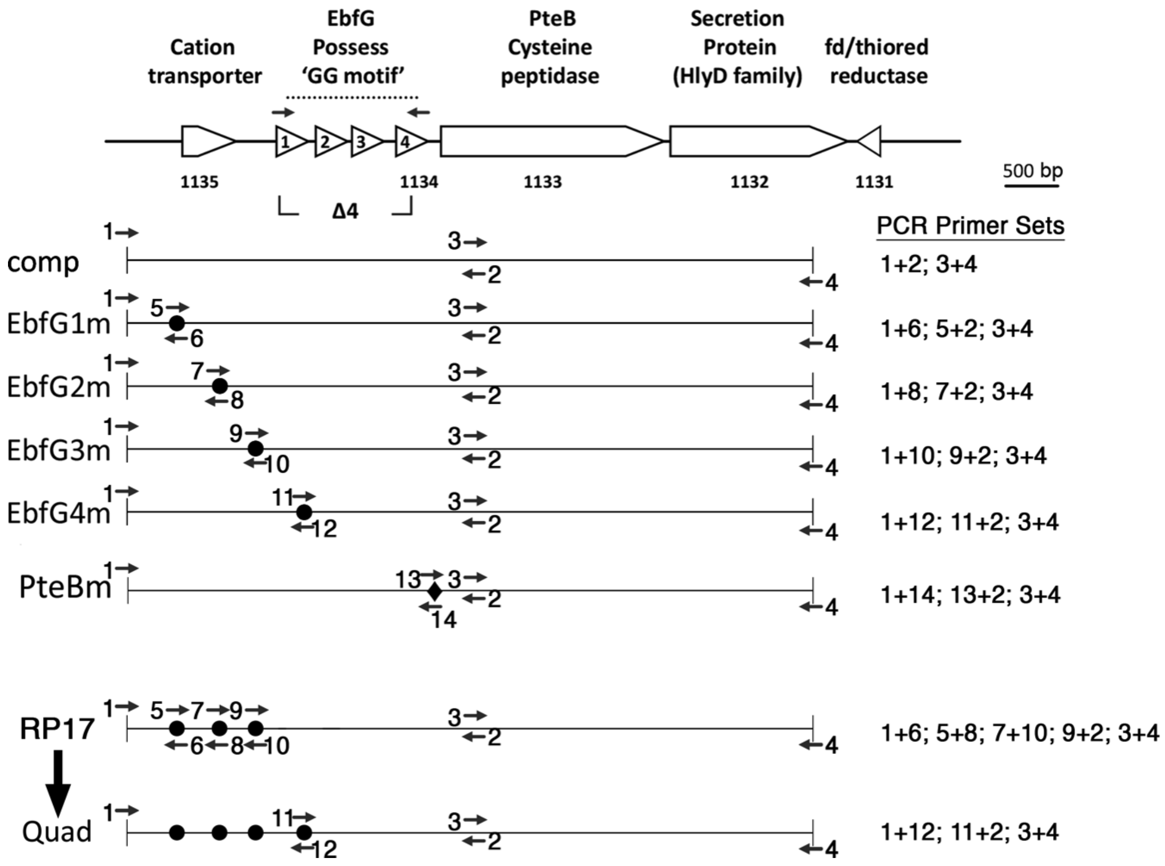

Figure S2: Generation of fragments for seamless assembly of complementation vectors. For each complementation vector, fragments were generated from wild type genomic DNA, or plasmid RP17 in the case of Quad fragment 1+12, using the indicated primers pairs. Introduced mutations are indicated by a dot or diamond as in Figures 1 and 3. Primers, listed in Table S2, are numbered as follows: 1) RP10-C2G-F, 2) RP10-R3, 3) RP10seq5, 4) RP10-G5C5-R, 5) ORF3GGmutAAF, 6) ORF3GGmutAAR, 7) ORF2GGmutAAF, 8) ORF2GGmutAAR, 9) ORF1GGmutAAF, 10) ORF1GGmutAAR, 11) 1134GAmutAA-F, 12) 1134GAmutAA-R, 13) 1133CmutA-F, and 14) 1133CmutA-R.
